# Supplementary material for: Protein S100-A7 Derived from Digested Dentin Is a Critical Molecule for Dentin Pulp Regeneration
Source: Cells. 2019 Aug 29;8(9):1002. doi: 10.3390/cells8091002 (PMC6769619; doi:10.3390/cells8091002)
Supplement: Supplementary file 1 [file cells-08-01002-s001.pdf]

Protein S100-A7 as a critical molecule for dentin pulp regeneration derived from digested

Shungo Komichi, Yusuke Takahashi, Motoki Okamoto, Manahil Ali, Masakatsu Watanabe,
Hailing Huang, Takeo Nakai, Paul Cooper, Mikako Hayashi

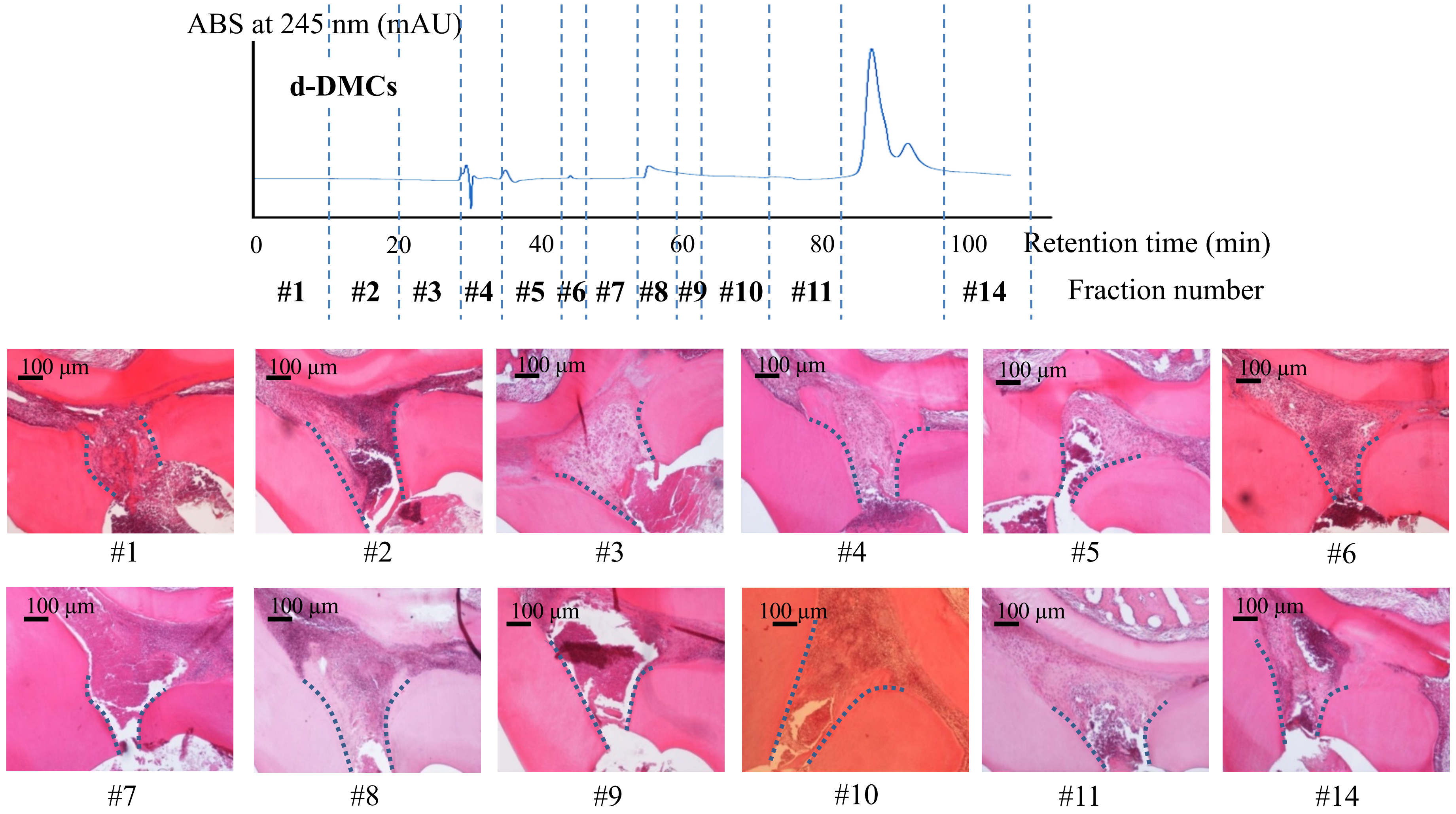

Supplementary Figure S1

Histological images of tertiary dentin using fractions #1–11 and #14 of d-DMCs.
No or little tertiary dentin was observed in all specimens (n=3).

Protein ID	Protein description	Protein ratio	Peptide PEP
tr|A0A024R1X8|A0A024R1X8_HUMAN	Junction plakoglobin	9999	0.005
sp|P31151|S10A7_HUMAN	Protein S100-A7	9999	1.095e-06
sp|P01834|IGKC_HUMAN	Ig kappa chain C region	9999	3.015e-04
tr|A0A0S2Z3E8|A0A0S2Z3E8_HUMAN	Fibrinogen alpha chain isoform 2	9999	9.417e-03
tr|F8W0P7|F8W0P7_HUMAN	ATP synthase subunit beta	3.345	3.213e-36
sp|P15924|DESP_HUMAN	Desmoplakin	1.076	2.000e-04
sp|Q02413|DSG1_HUMAN	Desmoglein-1	9999	4.041e-09
sp|Q6UWP8|SBSN_HUMAN	Suprabasin	9999	4.489e-07
tr|A8K9P0|A8K9P0_HUMAN	cDNA FLJ78413	14.17	7.7449e-06

Supplementary Table S1

Description of nine common proteins with higher abundance in d-DMCs

Protein ID	Protein description	Protein ratio	Peptide PEP
sp|P05109|S10A8_HUMAN	Protein S100-A8	in d-DMCs only	1.270e-27
sp|P12273|PIP_HUMAN	Prolactin-inducible protein	in d-DMCs only	0.036
sp|M0R1G4|YA044_HUMAN	Uncharacterized protein ENSP00000471857	in d-DMCs only	2.531e-06
sp|P02675|FIBB_HUMAN	Fibrinogen beta chain	in d-DMCs only	6.593e-07
sp|Q6KB66|K2C80_HUMAN	Keratin, type II cytoskeletal 80	in d-DMCs only	1.480e-04
sp|Q7RTS7|K2C74_HUMAN	Keratin, type II cytoskeletal 74	in d-DMCs only	2.035e-06
sp|Q8N1N4|K2C78_HUMAN	Keratin, type II cytoskeletal 78	in d-DMCs only	0.035
sp|Q9NZT1|CALL5_HUMAN	Calmodulin-like protein 5	in d-DMCs only	5.465e-04
tr|A0A068LN13|A0A068LN13_HUMAN	Ig heavy chain variable region	in d-DMCs only	7.485e-04
tr|A0N4V7|A0N4V7_HUMAN	HCG2039797	in d-DMCs only	0.010
tr|A7XZE4|A7XZE4_HUMAN	Beta tropomyosin isoform	in d-DMCs only	3.491e-04
tr|B4DKG7|B4DKG7_HUMAN	cDNA FLJ60633, highly similar to Homo sapiens secernin 3	in d-DMCs only	0.013
tr|E7EUT5|E7EUT5_HUMAN	Glyceraldehyde-3-phosphate dehydrogenase	in d-DMCs only	1.719e-03
tr|H0YBX3|H0YBX3_HUMAN	HUMAN Ribonuclease UK114	in d-DMCs only	2.119e-04
tr|H0YKX5|H0YKX5_HUMAN	Tropomyosin alpha-1 chain	in d-DMCs only	0.016
tr|K7EQW8|K7EQW8_HUMAN	Tropomyosin alpha-4 chain	in d-DMCs only	1.239e-04
tr|Q13707|Q13707_HUMAN	ACTA2 protein	in d-DMCs only	4.271e-04
tr|Q572P3|Q572P3_HUMAN	Tyrosine-protein kinase BTK isoform 52	in d-DMCs only	0.016
tr|Q6P670|Q6P670_HUMAN	TNNT3 protein	in d-DMCs only	9.196e-04
tr|Q9BWU5|Q9BWU5_HUMAN	Mutant hemoglobin beta chain	in d-DMCs only	0.006
tr|Q9P173|Q9P173_HUMAN	PRO2275	in d-DMCs only	7.072e-05
tr|U3PXP0|U3PXP0_HUMAN	Alpha globin chain	in d-DMCs only	0.019

Supplementary Table S2

Description of 22 proteins unique to d-DMCs
